# Chromatin accessibility profiling reveals that human fibroblasts respond to mechanical stimulation in a cell-specific manner

**DOI:** 10.1093/jbmrpl/ziae025

**Published:** 2024-02-29

**Authors:** Niall J Logan, Krystyna L Broda, Nikolaos Pantelireis, Greg Williams, Claire A Higgins

**Affiliations:** Department of Bioengineering, Imperial College London, London, SW7 2AZ, United Kingdom; Department of Bioengineering, Imperial College London, London, SW7 2AZ, United Kingdom; Department of Bioengineering, Imperial College London, London, SW7 2AZ, United Kingdom; Farjo Hair Institute, Manchester, M3 3EJ, United Kingdom; Department of Bioengineering, Imperial College London, London, SW7 2AZ, United Kingdom

**Keywords:** epigenetics, cell/tissue signaling, transcription factors, fibroblasts, ossification

## Abstract

Fibroblasts in the skin are highly heterogeneous, both in vivo and in vitro. One difference between follicular (dermal papilla fibroblasts [DP]) and interfollicular fibroblasts (papillary fibroblasts [PFi]) in vitro is their ability to differentiate in response to osteogenic media (OM), or mechanical stimulation. Here, we asked whether differences in the ability of DP and PFi to respond to differentiation stimuli are due to differences in chromatin accessibility. We performed chromatin accessibility and transcriptional profiling of DP and PFi in human skin, which arise from a common progenitor during development, yet display distinct characteristics in adult tissue and in vitro. We found that cells cultured in growth media had unique chromatin accessibility profiles; however, these profiles control similar functional networks. Upon introduction of a chemical perturbation (OM) to promote differentiation, we observed a divergence not only in the accessible chromatin signatures but also in the functional networks controlled by these signatures. The biggest divergence between DP and PFi was observed when we applied 2 perturbations to cells: growth in OM and mechanical stimulation (a shock wave [OMSW]). DP readily differentiate into bone in OMSW conditions, while PFi lack differentiation capability in vitro. In the DP we found a number of uniquely accessible promoters that controlled osteogenic interaction networks associated with bone and differentiation functions. Using ATAC-seq and RNA-seq we found that the combination of 2 stimuli (OMSW) could result in significant changes in chromatin accessibility associated with osteogenic differentiation, but only within the DP (capable of osteogenic differentiation). De novo motif analysis identified enrichment of motifs bound by the TEA domain (TEAD) family of transcription factors, and inter-cell comparisons (UpSet analysis) displayed large groups of genes to be unique to single cell types and conditions. Our results suggest that these 2 stimuli (OMSW) elicit cell-specific responses by modifying chromatin accessibility of osteogenic-related gene promoters.

## Introduction

Phenotypic behavior of a cell can be traced back to the genomic level where chromatin organization can influence accessibility to genomic regions and affect biological processes. In turn, chromatin organization and gene transcription can be influenced by external stimuli, such as temperature or light. A nice demonstration of this is found in Park et al,^1^ where cold stress was shown to lead to the degradation of the histone modification enzyme HDAC2, leading to an increase in histone acetylation and subsequent opening of chromatin and an increase in transcription. The advances in next-generation sequencing^2^ seen over the past decade now allow for epigenetic mapping of the genome and can be used to help answer fundamental questions relating to the role of chromatin organization in biological processes such as cell differentiation,^3–5^ development,^6^^,^^7^ and plasticity.^8^

Here, we applied these technologies to investigate differences between skin fibroblasts, which display both inter- and intra-location heterogeneity.^9^ Lineage-tracing studies have revealed that a fibroblast subtype found in the hair follicle, dermal papilla fibroblasts (DP), share a common developmental progenitor with an interfollicular subtype, papillary fibroblasts (PFi).^10^ Despite arising from the same developmental progenitor,^10^ DP and PFi have distinct identities both in vivo and in vitro; DP cells can differentiate down osteogenic^11^^,^^12^ and adipogenic^12^^,^^13^ lineages in vitro, while PFi lack this differentiation capacity.^14^ Dermal papilla fibroblasts and PFi also differ in their response to mechanical stimuli, and we previously demonstrated that mechanical stimulation of cells in culture (in the form of a single 165-kPa shock wave [SW] in air) in combination with osteogenic media (OM) resulted in enhanced and accelerated osteogenic differentiation of DP, whereas PFi were unresponsive to this stimuli.^14^ This accelerated osteogenic differentiation in response to a SW was observed at an epigenetic level (by DNA methylation status), at the transcriptomic level (increased *RUNX2* and *DLX5*), as well in functional assays (showing increased mineralization).^14^ It is important to note that the SW alone could not promote ossification of DP, but rather it acted in a synergistic manner with OM, accelerating and enhancing mineral deposition in follicular-derived DP cells.^14^ The use of DP cells (that can undergo osteogenic differentiation but would not normally do so in vivo) and matched sister cells, PFi cells (that cannot undergo osteogenic differentiation), provides a useful experimental model system for us to understand inappropriate ossification such as heterotopic ossification, which displays a high incidence following mechanical trauma, especially SW blast injury.

Based on the above, we hypothesized that external chemical stimuli, such as the introduction of osteogenic differentiation media, or mechanical stimuli, elicit a differential response in fibroblast subtypes due to differences in chromatin accessibility. To test this, we performed next-generation sequencing to assess both chromatin accessibility and transcriptional profiles in cells. Assay for transposase accessible chromatin in combination with high-throughput sequencing (ATAC-seq) is a technique that utilizes a genetically engineered hyperactive Tn5 transposase to tag and ligate fragments from regions of accessible chromatin throughout the genome.^15^ In combination with ATAC-seq we performed RNA-sequencing (RNA-seq) to evaluate if the accessibility of chromatin regions could be correlated with changes to transcriptional activity occurring in PFi and DP cells. Sequencing was initially performed on both fibroblast subtypes (DP and PFi) in growth media (GM) to establish differences between cells. Cells in growth media were considered as being in a “baseline” state. We then introduced 2 perturbations: a chemical one in the form of OM alone and a second one, which combined OM together with mechanical stimulation in the form of an SW (OMSW). While fibroblast subtypes displayed similarities in chromatin organization in GM, we found that the double perturbation (OMSW) resulted in a cell-specific response, with 2 distinct chromatin accessibility profiles with unique ontology signatures emerging. Specifically, within the DP cells, which we know have enhanced osteogenic differentiation capability in OMSW, we found that open chromatin was associated with an enrichment of osteogenic gene networks that were not present in any other cell type or condition. This indicates that chromatin rearrangements in response to external stimuli can occur in a cell-specific manner and helps explain the divergent response of DP and PFi to differentiation stimuli in vitro.

## Results

### PFi and DP cells share similar chromatin organization in GM conditions

Previous work showed that DP cells, but not PFi, will readily differentiate down an osteogenic lineage in vitro^14^; however, it is not known if this is due to baseline differences in the epigenetic landscape between each cell type. To ascertain the baseline landscape, and determine if DP are pre-sensitized to differentiate down an osteogenic lineage in vitro, we assessed both the chromatin accessibility and transcriptional profiles of each cell type in GM.

**Figure 1:**
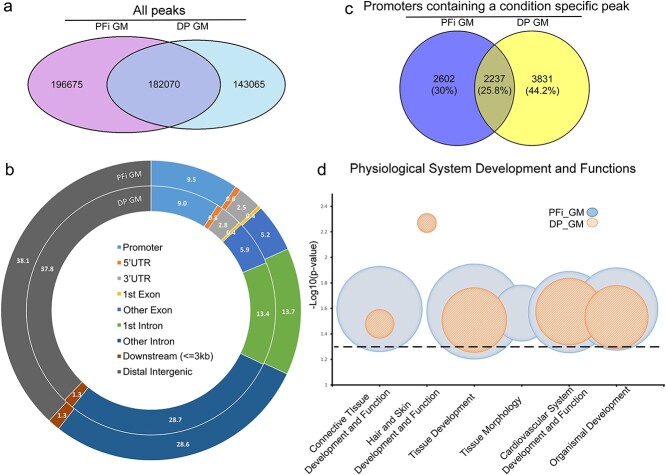
Growth media comparison of ATAC-seq data shows similarities between fibroblast subtypes: (a) Venn diagram displaying crossover of all ATAC peaks in each condition; (b) peak annotation of specific ATAC peaks on merged samples; (c) Venn diagram displaying gene promoters in each condition; (d) gene function analysis on ATAC data generated using IPA core analyses. The size of the bubble represents the number of molecules associated within that function. All physiological functions reported were deemed significant with p < .05 (dashed line represents the threshold). ATAC, assay for transposase accessible chromatin; DP, dermal papilla fibroblasts; GM, growth media; IPA, Ingenuity Pathway Analysis; PFi, papillary fibroblasts.

The ATAC-seq and RNA-seq libraries were generated from DP and PFi cells grown in GM, with nuclei and RNA isolated 48 hours after cell seeding (Supplementary Table S1). Early insert size analysis of ATAC-seq libraries showed the clear presence of a banding pattern, associated with nucleosome positioning of the transposase and correlating with high-quality libraries^15^ (Supplementary Figure S1). Post-sequencing quality checks showed an appropriate distribution of the number of called peaks between the biological replicates, which correlated with high fragments of reads in peaks (FRiP) scores greater than 0.35 (Supplementary Figure S2) and an enrichment of reads around transcription start sites (TSSs) (Supplementary Figure S3). Concordance correlation coefficients between the 2 sets of biological replicates were high, and reported as 0.96 and 0.88 for PFi and DP, respectively; therefore, replicates were merged for further analysis. A comparative analysis was then performed to identify peaks unique to either PFi or DP, referred to as condition-specific peaks. While 182 070 peaks were shared between the cell types, 196 675 peaks were unique to PFi and 143 065 were unique to DP cells (Figure 1a). As chromatin accessibility around TSS can affect transcription factor binding, this can ultimately lead to altered downstream transcriptional activity.^16^ We therefore decided to narrow our focus and filter the condition-specific peaks from each cell type, for peaks that were identified within 1000 bp of TSSs (referred to hereafter as promoter peaks). Peak annotation showed that the distribution of peaks was similar between the cell types (Figure 1b), with 9.0% and 9.5% of peaks falling within the promoter regions of PFi and DP cells, respectively. As multiple peaks can fall within a single promoter, we compared across each list to acquire gene lists of unique and shared promoters containing at least 1 condition-specific peak in either PFi or DP, identifying 2602 unique in PFi, 3831 unique in DP and 2237 shared between the two (Figure 1c). Using these lists, we performed an analysis using Ingenuity Pathway Analysis (IPA) core analyses (Qiagen). Despite identifying unique promoter peaks for each cell type, the core analysis revealed high similarities between cell types with regard to the types of networks in which these peaks were present (Figure 1d). Four of the top 5 “physiological system development and functions” identified were shared between DP and PFi, implying that, even though there are distinct chromatin accessibility landscapes in each cell type, the overcasting function of the genes regulated by the altered chromatin state was remarkably similar.

Using IPA to identify physiological system development and function terms overrepresented in either DP or PFi as described above, we identified only 1 unique function in DP cells, specifically associated with “hair and skin development and function.” This is of particular interest considering the known role that DP play in hair development and cycling. These cells, both in vivo and in vitro, are capable of instructing new hair follicle development, while PFi lack this capacity.^17^

**Figure 2:**
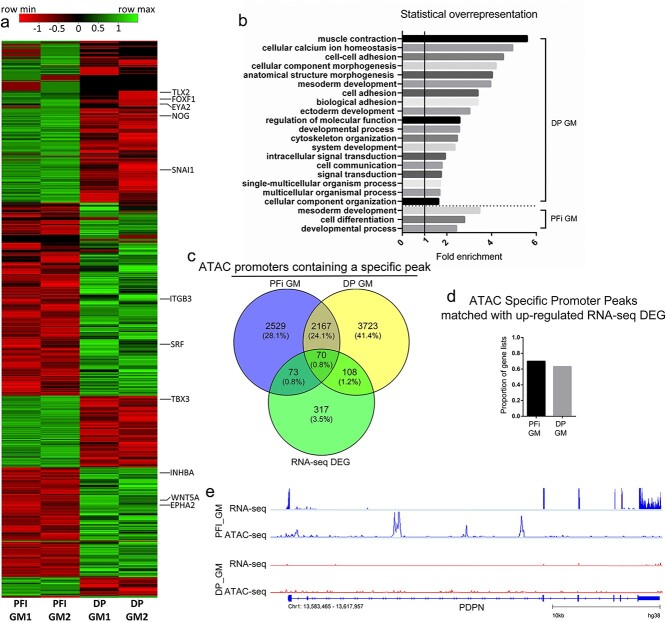
Growth media comparison of RNA-seq and ATAC-seq data shows similarities between fibroblast subtypes: (a) heatmap displaying DEGs from RNA-seq analysis (genes in the “mesoderm development” ontology term are shown); (b) GO terms showing statistically over-/underrepresented biological processes from upregulated genes in each cell type; (c) Venn displaying correlation of ATAC promoters and RNA-seq DEGs; (d) proportion of ATAC promoters that match with upregulated expression in RNA-seq data; (e) example of RNA-seq DEG matched with identification of an ATAC specific peak within its promoters. Gene shown: *PDPN*. ATAC, assay for transposase accessible chromatin; DP, dermal papilla fibroblasts; GM, growth media; PFi, papillary fibroblasts; GO, gene ontology; DEG, differentially expressed gene.

In addition to generating ATAC-seq data, we also performed RNA-seq analysis in order to obtain baseline transcriptional signatures for DP and PFi. Using the Bioconductor package DESeq2 to identify genes that were significantly (false discovery rate [FDR] ≤0.05) and differentially expressed (>2-fold) between the 2 cell types, we identified 310 genes upregulated in DP and 258 genes upregulated in PFi, compared with the other cell type (Figure 2a, Figure S4). We initially performed a core analysis with IPA and observed various metrics such as the percentage of genes within our lists that were found in “Canonical Pathways” (Supplementary Figure S5). However, we soon realized that a straightforward Gene Ontology (GO) assessment was more appropriate to identify the biological processes represented by the genes within DP or PFi. A GO analysis of upregulated genes in each cell type was then performed using the Panther Classification System, specifically using the “Panther overrepresentation test,” which analyses the representation of an ontology term in a given gene list compared with representation of that term in coding genes.^18^ For PFi, only 3 ontology terms were identified as overrepresented compared to 19 for DP (Figure 2b). Of the 3 PFi terms, 2 terms—“mesoderm development” and “developmental process”—were shared with DP, while only 1 term—“cell differentiation”—was unique. Within “mesoderm development,” genes including *WNT5A* and *ITGB3* were found in DP, while *SNAI1* and *FOXF1* were found in PFi (Figure 2a). The upregulated genes identified in DP cells had an overrepresentation of a variety of terms, including “cell adhesion” and “cytoskeletal organization” (Figure 2b). Notably lacking from the DP ontology analysis were any terms associated with osteogenic differentiation, suggesting that the DP cells are not primed to differentiate in response to the introduction of osteogenic media.

To ascertain if there was any correlation between transcriptional activity and chromatin accessibility in PFi and DP cells in GM, we cross-compared our RNA-seq gene lists and the ATAC-seq lists used in the earlier ontology analysis (Figure 2c). Of the 568 differentially expressed genes, 251 had been previously identified by our promoter-specific peak ATAC-seq analysis (Figure 1c). When we focused on the DP cells, 108 genes contained uniquely open chromatin in their promoters and also showed differential expression between DP and PFi. Of these, 63% of the genes were also upregulated in DP, indicating that enhanced transcriptional activity of these genes is associated with the presence of a condition-specific peak in their promoters (Figure 2d). With PFi, 73 genes that were differentially regulated at the transcriptional level also contained condition-specific ATAC-seq promoter peaks, with 70% of these showing an increase in RNA transcription. For example, podoplanin (*PDPN*), which is a well-described marker of PFi both in vivo and in vitro,^19^ has accessible chromatin uniquely in PFi, and is only transcribed in PFi (Figure 2e). These data demonstrate that, in general, the presence of a condition-specific ATAC peak within the promoter region of a gene is positively correlated with increased transcriptional activity.

We initially set out to determine baseline differences between DP and PFi, to help explain why we observe a differential response to perturbation of these cells in culture. Despite identifying unique chromatin accessibility profiles between cells, we found that the overarching physiological functions controlled by these open chromatin gene lists were relatively similar. This suggests that the differential response of cells in differentiation conditions is not due to baseline differences in chromatin architecture, and that DP cells are not epigenetically primed to differentiate into an osteogenic lineage.

### DP and PFi in OM start to diverge at the transcriptional level

Despite DP and PFi cells in GM each possessing a unique chromatin accessibility profile of gene promoters, our analysis so far demonstrated that the overcasting functions of these individual landscapes were, for the most part, overlapping. As both DP and PFi are fibroblast subtypes found in the skin dermis and originate from the same progenitor,^10^ similarities between these 2 cell populations is to be expected. However, as PFi cells do not share the same differentiation capacity as DP cells,^14^ we wanted to know if the introduction of a single chemical perturbation in the form of OM would result in differential changes in chromatin accessibility between fibroblast subtypes or if transcriptional changes alone are driving the osteogenic differentiation of DP cells.^11^^,^^12^

**Figure 3:**
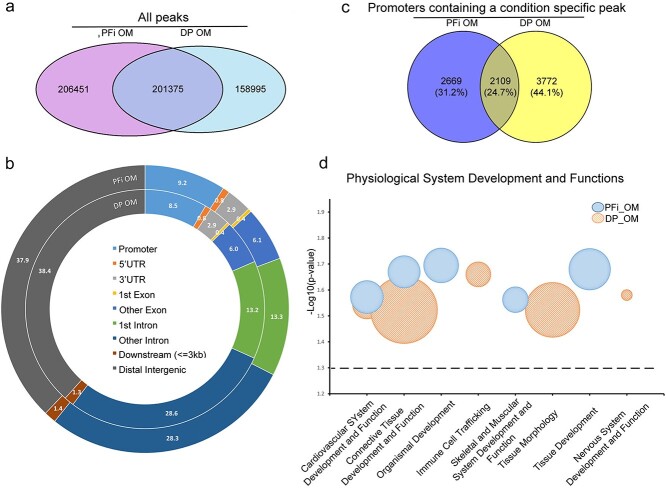
Osteogenic media comparison of ATAC-seq reveals divergence of physiological system development and functions: (a) Venn diagram displaying crossover of all ATAC peaks in each condition; (b) peak annotation of specific ATAC peaks on merged samples; (c) Venn diagram displaying gene promoters in each condition; (d) gene function analysis on ATAC data generated using IPA core analyses. The size of the bubble represents the number of molecules associated within that function. All physiological functions reported were deemed significant with p < .05 (dashed line represents the threshold). ATAC, assay for transposase accessible chromatin; DP, dermal papilla fibroblasts; IPA, Ingenuity Pathway Analysis; OM, osteogenic media; PFi, papillary fibroblasts.

To answer this, we performed ATAC-seq and RNA-seq on DP and PFi grown in OM for 24 hours. This early timepoint was selected as we wanted to identify changes associated with the initial introduction of OM, rather than changes associated with deposition of mineral (ossification), which occurs several days later. It would therefore be associated with cells undergoing osteoblastogenesis as opposed to osteogenesis. As with the GM ATAC libraries, a clear banding pattern^15^ was visible on all OM samples from insert size analysis (Supplementary Figure S1), while the number of called peaks was well distributed among biological replicate sets producing FRiP scores greater than 0.3 (Supplementary Figure S2). Biological replicates showed a high extent of concordance, with correlation coefficients reported as 0.92 and 0.93 for PFi and DP in OM, respectively, and were merged for further downstream analysis. A comparative analysis to identify condition-specific peaks found that 201 375 peaks were shared between both cell types, while 206 451 peaks were unique to PFi OM and 158 995 were unique to the DP OM condition (Figure 3a). Of these called peaks, 9.2% and 8.5% fell within the promoter region of PFi and DP, respectively (Figure 3b). After identifying genes associated with these promoters, we overlapped lists using a Venn diagram to acquire a list of 2669 genes unique to PFi, 3772 unique to DP, and 2109 shared (Figure 3c), and used the unique lists to perform gene function analysis in IPA. In contrast to the analysis performed on cells in GM, this time, only 2 of the top 5 physiological system development and functions identified were common to both cell types (Figure 3d). The other identified functions were unique to the top 5 list of either DP or PFi, suggesting that the chromatin accessibility profiles of cells are starting to diverge. While the physiological function “skeletal and muscular system development and function” was identified as representative of the PFi OM data, none of the identified functions for DP OM were related to osteogenic differentiation, suggesting that accessibility of chromatin may not be what is driving the osteogenic response of DP cells in OM.

**Figure 4:**
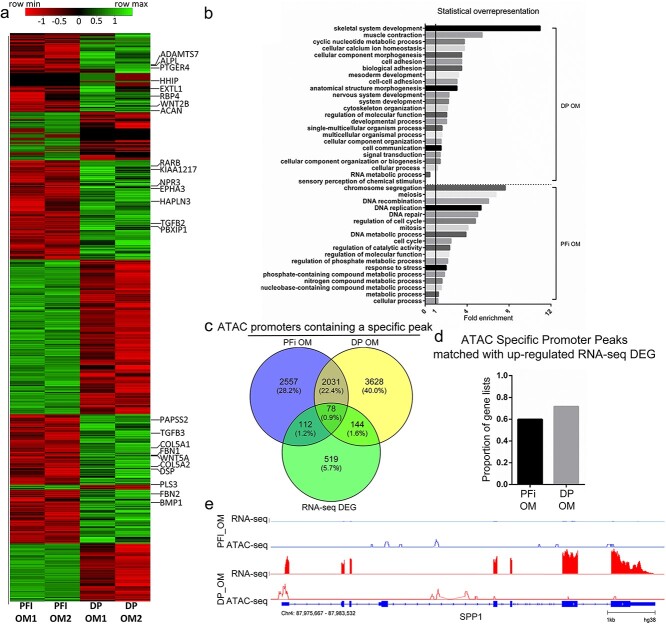
Osteogenic media comparison of RNA-seq reveals overrepresentation on skeletal differentiation terms in DP: (a) heatmap displaying DEGs from RNA-seq analysis (genes upregulated in DP OM in the “skeletal system development” ontology term are shown); (b) GO terms showing statistically over-/underrepresented biological processes from upregulated genes in each cell type; (c) Venn diagram displaying correlation of ATAC promoters and RNA-seq DEGs; (d) proportion of ATAC promoters that match with upregulated expression in RNA-seq data; (e) example of RNA-seq DEG matched with identification of an ATAC specific peak within its promoters. Gene shown*: SPP1.* ATAC, assay for transposase accessible chromatin; DP, dermal papilla fibroblasts; OM, osteogenic media; PFi, papillary fibroblasts; GO, gene ontology; DEG, differentially expressed gene.

In addition to the ATAC analysis, RNA-seq was performed on DP and PFi in OM, enabling identification of 455 and 398 genes that were upregulated, respectively, in DP and PFi (Figure 4a, Figure S4). We conducted a canonical pathway analysis with IPA (Figure S5) and a statistical overrepresentation test using Panther.^18^ With Panther, we found 22 terms overrepresented in the DP OM gene list. This included 17 of the 19 terms previously identified in the DP GM list (Figure 2b), suggesting identification of a DP-specific gene signature regardless of the culture media. Intriguingly, the most significantly overrepresented ontology term in the DP OM (Figure 4b) was “skeletal system development,” which has daughter terms including “bone cell development,” “ossification involved in bone maturation,” and “mesenchymal cell differentiation involved in bone development.” Genes, including *COL5A2*, *COL1A1*, *ALPL*, *HAPLN3*, *TGFβ2*, and *ACAN*, were upregulated in DP OM versus PFi OM and were associated with the “skeletal system development” ontology term (Figure 4a). In PFi OM, 18 ontology terms were also overrepresented, including a number associated with “metabolic processes,” “cell cycle,” and “DNA replication.” Despite both the PFi and DP cells being grown in OM for 24 hours, there were no terms associated with osteogenic differentiation identified in the PFi dataset.

Last, we wanted to assess if there was any correlation between our ATAC-seq OM chromatin accessibility profiles and our OM differentially expressed gene lists. We found that, of 853 differentially expressed genes, 334 were also identified as having condition-specific open chromatin at their promoters (Figure 4c). Similar to the GM analysis, of the genes that did correlate between RNA and ATAC lists, we found that 60% and 72% of genes, respectively, in PFi and DP showed an upregulation in transcriptional activity with the presence of a condition-specific peak located within the promoter region of the target gene (Figure 4d). For example, we found that osteopontin, also known as secreted phosphoprotein 1 (*OPN, SPP1*) was not only upregulated by 3.4-fold in DP cells compared with PFi but that it also had a condition-specific peak within its promoter (Figure 4e). SPP1 has a well-known role in bone formation and bone marrow mesenchymal stem cells, with *Spp1*-/- mice demonstrating impaired bone formation both in vitro and in vivo.^20^ As PFi cells do not mineralize in OM, this effect could possibly be driven by inaccessible chromatin around the *SPP1* TSS, leading to an inability of upstream transcription factors to bind and activate gene transcription. Perhaps highlighting the caution that should be taken in interpreting gene ontology data, *SPP1* was not identified in the “skeletal system development” ontology term, despite having a key role in osteogenesis. Instead, *SPP1* is represented by “biological process” GO terms including “osteoblast differentiation” and “ossification.”

Analysis of the ATAC-seq data of both PFi and DP in OM demonstrated that the fibroblast subtypes have unique chromatin accessibility profiles around TSSs, which, following ontology analysis, revealed the emergence of 2 distinct epigenetic landscapes. The epigenetic landscape of both PFi or DP failed to demonstrate any enrichment towards an osteogenic cell phenotype, which suggests that the chromatin organization of PFi and DP in OM may not be driving the osteogenic differentiation of DP cells in OM. Interestingly, ontology analysis of the RNA-seq data revealed a prominent overrepresentation of the GO term “skeletal system development” in DP cells, suggesting that the transcriptional profile of DP cells in OM is more osteogenic than PFi.

### Cell-specific epigenetic landscapes arise in PFi and DP cells exposed to OMSW

When fibroblast subtypes were grown in OM, we observed the emergence of an RNA transcriptome in DP cells with an osteogenic signature. However, the ATAC-seq profiling did not reveal any changes in chromatin indicating an epigenetic response of cells to the OM. We have previously shown that application of a single 165-kPa SW results in a synergistic acceleration and enhancement of mineralization in DP cells in OM, yet has no effect on PFi.^14^ We therefore decided to investigate whether these 2 perturbing stimuli (a SW together with OM) might result in chromatin reorganization in a cell-specific manner.

**Figure 5:**
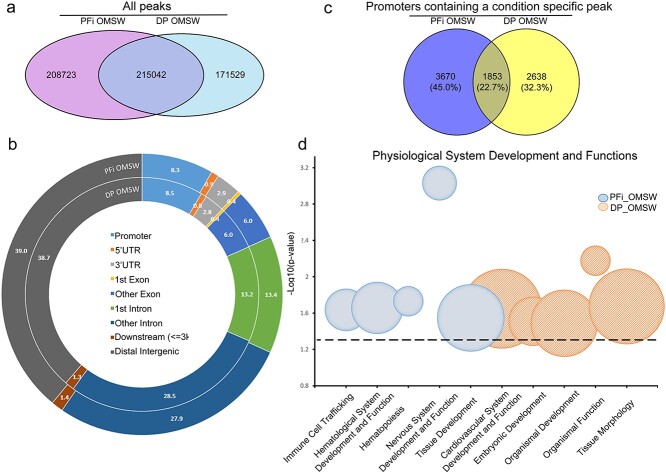
An OMSW comparison uncovers unique chromatin signatures in fibroblasts: (a) Venn diagram displaying crossover of all ATAC peaks in each condition; (b) peak annotation of specific ATAC peaks on merged samples; (c) Venn diagram displaying gene promoters in each condition; (d) gene function analysis on ATAC data generated using IPA core analyses. The size of the bubble represents the number of molecules associated within that function. All physiological functions reported were deemed significant with p < .05 (dashed line represents the threshold). ATAC, assay for transposase accessible chromatin; DP, dermal papilla fibroblasts; IPA, Ingenuity Pathway Analysis; OMSW, osteogenic media together with mechanical stimulation in the form of a shock wave; PFi, papillary fibroblasts.

To evaluate this, DP and PFi cells were exposed to a single 165-kPa SW using a custom-built shock tube.^21^ Immediately after this, OM was added to the cells and, 24 hours later, nuclei and RNA were isolated to generate ATAC and RNA-seq libraries. As before, ATAC libraries showed clear banding patterns (Supplementary Figure S1), the number of called peaks was well distributed between biological replicates, and FRiP scores were higher than 0.3 (Supplementary Figure S2). Concordance correlation coefficients between replicates were 0.9 and 0.93 for PFi OMSW and DP OMSW, respectively, and sets were merged for further analysis. The same comparative analysis was performed as in GM and OM to identify condition-specific peaks, identifying 215 042 shared peaks, 208 723 peaks unique to PFi OMSW, and 171 529 peaks unique to DP OMSW (Figure 5a). Of these, 8.3% and 8.5% fell within a promoter region in PFi and DP cells (Figure 5b), respectively. A Venn analysis of these data revealed 3670 unique gene promoters with a condition-specific peak in PFi and 2638 in DP (Figure 5c). A core analysis of these gene lists, described as chromatin accessibility profiles, was performed in IPA to identify 5 top physiological system development and functions for PFi and DP in OMSW. In contrast to the GM core analysis, which showed a high amount of overlap between PFi and DP in GM, exposure to 2 perturbing stimuli in the form of OMSW appears to generate distinct and unique ontology signatures, with no overlap between DP and PFi cells (Figure 5d).

**Figure 6:**
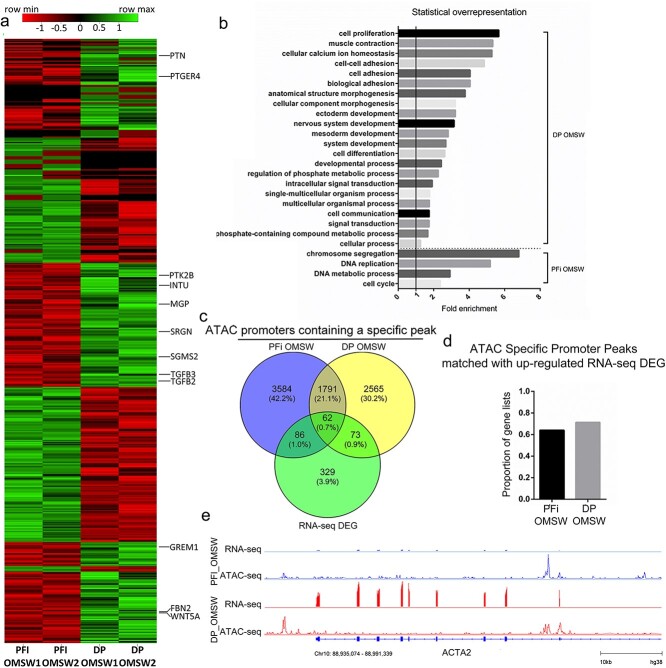
An OMSW comparison uncovers unique chromatin signatures in fibroblasts: (a) heatmap displaying DEGs from RNA-seq analysis (genes upregulated in DP OMSW from the ontology term “regulation of ossification” are shown); (b) GO terms showing statistically over-/underrepresented biological processes from upregulated genes in each cell type; (c) Venn diagram displaying correlation of ATAC promoters and RNA-seq DEGs; (d) proportion of ATAC promoters that match with upregulated expression in RNA-seq data; (e) example of RNA-seq DEG matched with identification of an ATAC specific peak within its promoters. Gene shown: *ACTA2*. ATAC, assay for transposase accessible chromatin; DP, dermal papilla fibroblasts; OMSW, osteogenic media together with mechanical stimulation in the form of a shock wave; PFi, papillary fibroblasts; GO, gene ontology; DEG, differentially expressed gene.

To determine if the combination of OM together with a SW also resulted in distinct transcriptional signatures, we performed RNA-seq analysis, identifying 290 and 260 genes significantly upregulated, respectively, in DP and PFi cells in OMSW (Figure 6a, Figure S4). A canonical pathway analysis in IPA (Supplementary Figure S5) was conducted in addition to a GO analysis using an “overrepresentation test” in Panther,^18^ revealing 4 overrepresented terms in PFi, and 22 in DP (Figure 6b). In PFi OMSW, all 4 of the overrepresented terms were also identified in the PFi OM ontology analysis (Figure 6b), suggesting that the SW has little effect on transcriptional activity in PFi. In the DP OMSW analysis, 8 of the 22 terms, including “cell proliferation” and “cell differentiation,” were new to this analysis compared with the DP OM. Collectively, 7 of the terms in DP OMSW were associated with development or morphogenesis of some description, while 3 were associated with cell adhesion, suggesting that these biological processes are important for differentiation of DP in OMSW. Within the broad term “multicellular organismal process” there are daughter terms including “regulation of bone mineralization” and “regulation of ossification.” There were 12 genes within the DP OMSW gene list found within the “regulation of ossification” GO term (fold enrichment, 7.73), 8 of which were also within the “regulation of bone mineralization” term (fold enrichment, 7.70). This included genes such as *MGP*, *TGFB2*, and *TGFB3*, with well-known roles in promoting ossification (Figure 6a).

As in previous comparisons, we also wanted to correlate our chromatin accessibility profile lists and our RNA-seq differentially expressed gene lists to see if chromatin rearrangements in promoters were affecting transcription. Comparing the lists demonstrated some crossover between the 2 datasets, with 221 out of 550 genes showing overlap (Figure 6c). Of these, similar rates of directional transcription were observed, with 64% and 71% of genes in PFi and DP OMSW (Figure 6d), respectively, showing enhanced transcriptional activity with the presence of a condition-specific peak located within the promoter region of the target gene, as shown in the example of actin alpha 2 smooth muscle (*ACTA2*) *(*Figure 6e).

Using ATAC and RNA-seq, we found that DP and PFi exposed to OMSW resulted in the formation of 2 distinct epigenetic landscapes, containing unique cell-specific chromatin accessibility profiles. Overrepresentation analysis of the upregulated genes in PFi OMSW showed no evidence of differentiation terms, while genes in DP OMSW were overrepresented for “cell differentiation” (Figure 6b). Assessing the correlation between condition-specific ATAC-seq promoters and RNA-seq gene lists we found that a sizable proportion of differentially expressed genes also have increased promoter accessibility. The remaining differentially expressed genes are likely influenced by changes in chromatin accessibility outside of promoters, such as in introns, or enhancers, which can also regulate gene expression.

### Motif analysis to infer possible upstream regulators identifies TEAD

**Figure 7:**
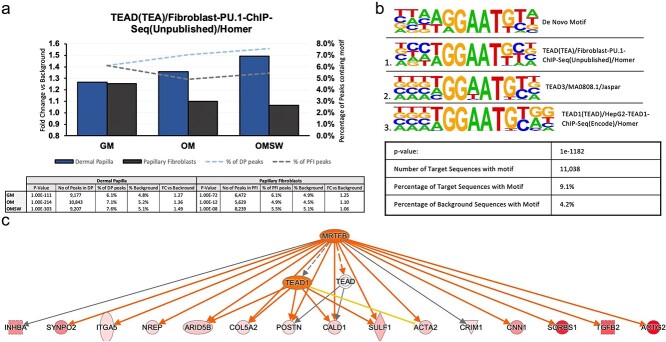
TEA domain (TEAD) family of transcription factors identified as possible upstream regulators of DP OMSW signature: (a) known enrichment of generic TEAD family motif shows increasing enrichment of TEAD motif in DP with change from GM to OM to OMSW while this enrichment is not observed in PFi; (b) de novo motif analysis identifies strong enrichment in motif that closely matches TEAD family motif sequence; (c) IPA analysis carried out on upregulated DP OMSW genes highlights mechanistic network of *MRTFB* and *TEAD1* as one of the top upstream regulator pathways. DP, dermal papilla fibroblasts; GM, growth media; IPA, Ingenuity Pathway Analysis; OM, osteogenic media; OMSW, osteogenic media together with mechanical stimulation in the form of a shock wave; PFi, papillary fibroblasts.

While unique ATAC-seq peaks within the promoter region of differentially expressed genes suggest that epigenetic changes are occurring upstream of gene expression, it is uncertain what the mechanism underlying these observed changes is. To try and answer this question, we used Hypergeometric Optimization of Motif EnRichment (HOMER) to identify motif sequences that were overrepresented within these peaks and combined this with predicted upstream regulator analysis in IPA using differentially expressed genes between DP OMSW and PFI OMSW (Figure 7).

We first looked at known motif enrichment in OMSW conditions. Low-incidence motifs (<5% of target sequences and <1.2 fold change (FC) vs background) and those present in PFi OMSW were filtered out, revealing that the top 5 most enriched motifs were all members of the TEA domain (TEAD) family (Supplementary Table S2). When we evaluated the incidence of TEAD motifs across cell types and conditions we found that TEAD motifs appear to increase in enrichment within DP from GM to OM to OMSW, implying that epigenetic changes associated with more potent osteogenesis occur at TEAD regulated loci (Figure 7a).

To corroborate what was seen in known motif enrichment, de novo motif analysis was carried out whereby motifs are assembled by HOMER without the use of reference motifs that may add bias. This analysis further highlighted TEAD, with the top scoring unique motif (also not present in PFi OMSW), showing a near-exact match to TEAD and present in 9.1% of the DP OMSW target sequences (Figure 7b).

Concomitantly, we also performed an analysis in IPA using the RNA-seq data to identify potential upstream regulators of the DP OMSW transcriptional signature. With this, TEAD was also implicated in the gain of osteogenic potency, with 4 TEAD family factors (TEAD3, TEAD1, TEAD4, and TEAD2) and 3 factors directly related to TEAD - Myocardin Related Transcription Factor B (MRTFB), Serum Response Factor (SRF), and Myogenic Differentiation 1 (MYOD) - predicted to regulate DP OMSW differentially expressed genes (Supplementary Table S3).

Of these TEAD-related factors, MRTFB was ranked third overall in upstream transcriptional regulators (Benjamini-Hochberg (BH)-corrected p-value, 4.98 × 10^-7^, and *z*-score activation, 3.571). In the predicted mechanistic network related to MRTFB, the TEAD family and TEAD1 were linked as plausible downstream targets of MRTFB (Figure 7c).

### Multi-way comparative analysis of chromatin organization reveals DP OMSW are enriched towards an osteogenic identity

The RNA-seq analysis gave us lists of genes expressed within both DP and PFi in GM, OM, and OMSW conditions although we specifically collected RNA at early time points, prior to any osteogenesis occuring. To see if there was an increase in “osteogenic genes” within cells in OMSW highlighting an early transition to a differentiating state, we looked for the percentage of overlap of genes in our datasets compared with a list of genes published by Hakelien et al^22^ known to be differentially expressed in human mesenchymal stem cells (MSCs) after 28 days in OM. This analysis revealed that, at the transcriptional level, there were minimal differences between cells in GM, OM, or OMSW, 24 hours after exposure to the specific condition (Supplementary Figure S6).

Our previous analysis of the ATAC-seq data was designed to identify cell-specific changes in chromatin accessibility, as DP and PFi were compared with one another in similar conditions (either GM, OM, or OMSW). However, this analysis did not give us insight into the chromatin rearrangements that occur within a cell type as a result of a specific perturbation. Instead, in our inter-cell comparisons of chromatin accessibility, we identified lists of genes that contained a condition-specific peak within their promoter region unique to the cell type in that comparison. To narrow our focus, and acquire lists that contained genes unique to both a cell type and a specific perturbation, we evaluated these inter-cell comparison lists by performing a 6-way UpSet analysis.^23^ Visualization of the UpSet intersection analysis^24^ showed that, while some genes were shared between cell types and across comparisons, the largest groups of genes were those unique to a single cell type and condition (Figure 7a).

When we initiated this work, we postulated that cell-specific epigenetic differences were enabling DP cells, but not PFi, to mineralize in vitro when cultured in OM. As SW exposure could enhance mineralization,^14^ we specifically wanted to analyze the effect of an SW on DP chromatin organization. Using the unique lists exported from our 6-way UpSet analysis, we performed IPA ontology of the genes found only within DP cells in the OMSW comparison, identifying multiple networks strongly associated with osteogenic functions (Figure 7b). The top network generated in core analysis from IPA in DP OMSW showed interactions of our identified genes with a number of osteogenic master regulators, such as Runt-related transcription factor 2 (*RUNX2*)^25^ and Osterix (*SP7*)^26^ (Figure 7c), despite these master regulators themselves not being upregulated in the DP OMSW gene list. Notably, inclusion and predicted activation of integrin alpha V (*ITGAV*) was also observed within the network (Figure 7c) in addition to being significantly upregulated in the DP OMSW versus PFi OMSW RNA-seq data. *ITGAV* is a cell-surface mechano-sensor, which increases in expression in MSCs undergoing osteogenic differentiation,^27^ while knockdown can lead to adipogenic differentiation in adipose stem cells.^28^ We previously identified *ITGAV* as a biomarker of OMSW-induced ectopic mineralization (in bisulfite sequencing data), and demonstrated that *ITGAV* inhibition can abrogate OMSW-induced mineral deposition in DP cells in vitro.^14^ The inclusion and predicted activation of *ITGAV* within the top pathway analysis network generated from chromatin accessible unique DP OMSW genes strengthens our hypothesis that *ITGAV* is a key player in OMSW-induced ossification. To assess if the incorporation of *ITGAV* and other osteogenic-associated genes into the top networks generated in IPA was specific, or an artefact of IPA, we also assessed the top networks generated in IPA from the other 5 unique gene lists (PFi OMSW, DP OM, PFi OM, DP GM, PFi GM) from our 6-way Venn analysis. We found that no other gene lists, including the DP OM list, resulted in generation of IPA networks containing a single osteogenic-associated function (Figure 7b). These data imply that, within the OMSW comparison, DP cells have unique chromatin accessibility at gene promoters enabling enhanced osteogenic differentiation. Considering the absence of osteogenic-associated networks generated from chromatin accessibility lists from DP OM, which do ossify, albeit slower than DP OMSW, our analysis suggests that restructuring of chromatin occurs as a result of SW exposure in a cell-specific manner. We propose that this leads to the formation of an epigenetic landscape enriched towards osteogenic differentiation and helps to explain the accelerated ossification observed in DP cells in response to SW exposure.

## Discussion

In this body of work, we set out to decipher why DP cells can differentiate down an osteogenic lineage, while their sister cell type (PFi) are unresponsive to osteogenic stimuli. This DP differentiation in vitro does not have physiological relevance as it does not occur in vivo; however, we believe that this makes DP a good cell type to study inappropriate ossification, or heterotopic ossification in vitro. For this reason, we used a mechanical stimulus (SW) to promote ossification that replicated the trauma stimulus which induces heterotopic ossification in vivo.^14^ We hypothesized that inherent (baseline) differences in the epigenetic status of these 2 fibroblast subtypes (in GM), DP and PFi, were enabling this differential response (in OM and OMSW). However, using ATAC-seq to evaluate regions of open chromatin, we found that the chromatin accessibility profile of both DP and PFi in GM results in activation of genes with comparable ontological networks, suggesting inherent similarities in the chromatin architecture between the cell types. When perturbing stimuli that enhance ossification of DP cells, but not PFi, were introduced, we gradually observed a divergence of the epigenetic signatures of the fibroblast subtypes, suggesting that the stimuli itself elicit a cell-specific response. Specifically, we found that, when DP cells are exposed to both mechanical stimulation (a SW) and chemical stimulation (OM), which we know can enhance and accelerate ossification of DP cells,^14^ they acquire a unique chromatin profile of accessible promoters at genes associated with osteogenic differentiation (Figure 7b, c). As samples were analyzed just 24 hours after exposure to OMSW, several days before osteogenic differentiation actually occurs, it suggests that these chromatin rearrangements occur as a result of exposure to stimuli, which leads to a trend towards osteoblastogenesis, rather than occurring as a result of osteogenic differentiation. This makes it even more intriguing that PFi do not show the same rearrangements, highlighting that we are observing not only a differential ability of DP and PFi to ossify but a cell-specific chromatin rearrangement response to mechanical and chemical perturbations. We specifically selected a 24-hour timepoint for analysis as we wanted to identify changes prior to differentiation. A time-course analysis would enable us to answer questions different from those addressed here, such as is chromatin in a repressed state early on, but becomes permissive in response to modification of chromatin remodeling enzymes or pioneer transcription factors?

The effect of chromatin accessibility on cell differentiation is now beginning to be investigated fully and reported in the literature thanks to recent advances in genomic-sequencing technologies. Using DNAse 1 hypersensitivity to assess a human fetal osteoblastic cell line (hFOB), Thompson et al^29^ detected large-scale reorganization of the chromatin landscape upon osteogenic induction, with clear changes present between cells in basal media or OM after 48 hours of differentiation. To identify potential transcription factors that may be binding promoters and affecting the differentiation of hFOBs, motif analysis was performed and revealed that cells in OM had accessible chromatin at binding motifs for the osteogenic master regulator *RUNX2*.^29^ In our work, the motif and master regulator analysis both identified TEAD family members as potential regulators of ossification. TEADs are transcription factors known to form complexes with Yes-associated protein (YAP) and transcriptional co-activator with a PDZ-binding motif (TAZ) to facilitate binding to DNA in the regulation of gene expression.^30^ TEAD2 has previously been proposed as a novel regulator of osteogenic differentiation by the discovery of chromatin alterations within its promoter region in MSCs undergoing osteogenic differentiation.^22^ Within the same study, knockdown of TEAD2 using siRNA had a negative effect on mineralization, implying that TEAD2 plays a significant role in later stages of osteogenic differentiation. The transcriptional co-activators YAP/TAZ have also been shown to have important roles in osteogenic differentiation,^31–33^ while inhibition of the YAP/TAZ reaction with TEAD using small molecules can reduce osteogenic gene expression.^34^

In DP cells exposed to perturbation with OMSW, we identified changes occurring to the chromatin epigenetic landscape that were associated with enhancement of ossification. It has previously been proposed that application of mechanical forces can lead to both short-term and long-term changes to chromatin function. Short-term responses, described as a mechano-response, can lead to chromatin decondensation at force-selective genes that could be a means to dissipate mechanical stress.^35^ It is thought that outside-in signaling via cell-surface integrins can propagate stresses to the actin cytoskeleton, resulting in changes to nuclear lamins and subsequently chromatin decondensation.^36^ How these mechanical stresses are transferred from nuclear lamins to chromatin is likely affected by chromosomal locations within the nucleus,^37^ and this positioning may explain cell-specific responses to mechanical forces. Effects from long-duration mechanical forces, described as mechano-adaption,^35^ can lead to chromatin compaction,^38^ highlighting the ability as well as the complexity of the effect of mechanical stimulation on cell response.

**Figure 8:**
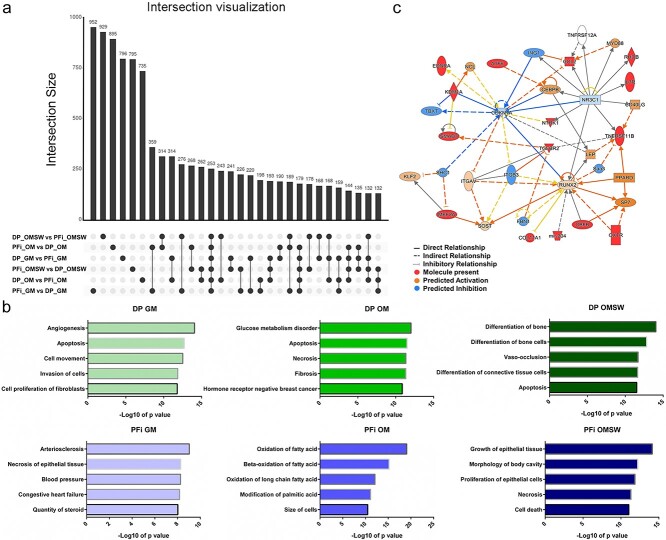
Comparative analysis shows osteogenic enrichment in DP OMSW chromatin profile: (a) intersection visualization analysis showing the presence of a high number of unique genes in each cell type within each comparison; (b) IPA-generated network from the unique DP OMSW gene list taken from intersection analysis, showing osteogenic-associated interactions; (c) comparison of the top 5 functions associated with the top generated IPA network created using intersection analysis unique gene lists. Only DP OMSW was found to possess osteogenic associated chromatin networks. DP, dermal papilla fibroblasts; GM, growth media; IPA, Ingenuity Pathway Analysis; OM, osteogenic media; OMSW, osteogenic media together with mechanical stimulation in the form of a shock wave; PFi, papillary fibroblasts.

Here, we observed that a mechanical stimulus, in the form of an SW, could result in changes in chromatin accessibility in a cell-specific manner, leading to enhanced osteogenic differentiation. We have previously shown that another epigenetic mechanism, DNA methylation, is also altered in DP and human bone marrow MSCs in OMSW.^14^ One of the genes whose promotor was hypo-methylated as a result of SW exposure in OM was the osteogenic-associated^27^ mechano-sensor *ITGAV*, which was also identified as activated within the top chromatin accessibility network from DP cells in OMSW in this current study (Figure 8b). Expression of *ITGAV* is also significantly increased in DP OMSW mRNA versus PFi cells in OMSW (Figure 6a,b), which notably are unable to mineralize. In summary, we have demonstrated both changes to chromatin accessibility (Figure 8bc) and DNA methylation^14^ of *ITGAV* and other genes, which occur in a cell-specific manner in response to perturbation with OMSW, resulting in enhanced ossification in vitro.

To conclude, in this body of work we used ATAC-seq combined with RNA-seq to demonstrate that exposure to a mechanical stimulus (SW) together with a chemical stimulus (OM) elicits a cell-specific response in human DP cells, modifying chromatin accessibility and enabling accelerated differentiation down an osteogenic lineage. These findings support our assertion that certain cell types are more responsive to mechanical stimuli (SW) that induce heterotopic ossification. Next, we hope to determine how this occurs and identify cell-specific therapeutic targets to modulate bone formation after SW injury.

## Materials and methods

### Study design

Here, we performed a combination of ATAC-seq and RNA-seq on human DP and PFi cells cultured in GM or OM only, or OMSW. The main aim of this was to assess the role of chromatin structure in osteogenic differentiation and determine if certain cell types are sensitized to differentiation down an osteogenic linage. We performed our experiments in 3 stages:

1. First, we gathered nuclei (for DNA) and RNA for sequencing from both DP and PFi cells, in the following conditions; GM, OM, and OM plus a 165-kPa SW (OMSW).

2. Using both the nuclei and RNA gathered in step (1) we performed ATAC library preparation for sequencing following a previously published protocol^15^^,^^39^ and RNA-seq using Smart-seq2 library preparation.^40^

3. We then completed bioinformatics analysis on ATAC libraries using an esATAC R script, and RNA libraries with SeqMonk. Three inter-cell comparative analyses were performed, first by comparing PFi and DP in GM, followed by PFi and DP in OM, and last PFi and DP in OMSW comparison. We generated lists containing differentially expressed genes or condition-specific ATAC peaks, and analyzed downstream functions controlled by these genes in IPA. Differentially expressed genes generated from RNA-seq data were validated using RT-quantitative PCR (RT-qPCR).

4. Last, we performed comparative analysis between our ATAC lists from each comparison using UpSet and InteractiVenn, revealing the relationship between chromatin structure and how it can sensitize cells towards an osteogenic identity. The sample sizes for our in vitro tests were taken from similar studies reported in the literature. The exact number for each experiment can be found in the figure legends. Investigators were not blinded when conducting or evaluating the experiments.

### Cell isolation and culture

The DP and PFi cells were isolated from discarded tissue from patients undergoing hair transplant surgeries after written informed consent was obtained (patient demographics detailed in Table 1). Microdissection techniques were used to isolate both DP and PFi from the tissue, as previously described.^41^ Cells were cultured in GM that consisted of DMEM (ThermoFisher, 61 965-026) supplemented with 10% FBS and 1% penicillin/streptomycin (P/S; ThermoFisher, 15 070-063). Osteogenic media consisted of low-glucose DMEM (LG-DMEMl ThermoFisher, 31 885-023) containing 10% FBS, 1% P/S, 100 nM dexamethasone (Sigma Aldrich, D4902), 50 μM L-ascorbic acid 2-phosphate (Sigma Aldrich, A8960), and 10 mM β-glycerol phosphate (Sigma Aldrich, G9422).

**Table 1:** Details of cells used.

**Patient ID**	**Gender**	**Age, y**	**Cell populations**
1	M	26	DP and PFi (matched)
2	M	52	DP and PFi (matched)

Abbreviations: DP, dermal papilla fibroblasts; M, male; PFi, papillary fibroblasts.

### SW exposure

The DP and PFi cells were seeded into 35-mm Petri dishes at 7 × 10^4^ cells per dish and left overnight in standard culture conditions of 37°C, 5% CO_2_ in a humidified environment. The following day, using a compressed air-driven shock tube, cells were exposed to one 165-kPa SW, as previously described.^14^^,^^21^ Medium was changed to either GM or OM immediately following SW exposure. Twenty-four hours post-SW exposure, cells were harvested to be used for nuclei or RNA isolation.

### RNA-seq

The DP and PFi cells in GM, OM, and OMSW from 2 biological replicates were homogenized by centrifugation through Qiashredders (Qiagen, 79 654), and total RNA was isolated using a commercially available kit, as per the manufacturer’s instructions (Qiagen, RNeasy Plus Micro Kit, 74 034). Quality control was performed in the form of a Qubit RNA high-sensitivity assay (ThermoFisher, Q32852), while an Agilent Technologies Bioanalyzer was used to attain RNA integrity (RIN) scores. All samples had RIN numbers greater than 9. cDNA library construction was performed at Oxford Genomics (Oxford, United Kingdom) using the Smart-seq2 library preparation protocol,^40^ and libraries were sequenced using 75-bp paired-end-reads on 2 lanes of an Illunmia HiSeq 4000 instrument. Samples were re-pooled when running the second lane to generate libraries of approximately equal size.

### ATAC-seq

Libraries for ATAC-seq were generated by following a previously reported method.^15^^,^^39^ The DP and PFi cells in GM, OM, and OMSW from 2 biological replicates were washed twice in ice-cold PBS and incubated with 0.5% trypsin–EDTA for 5 minutes until the cells dissociated from the dish. Media was then added to the cells, which were centrifuged at 300 *g* for 4 minutes to form a pellet. Each pellet was re-suspended in fresh media, counted, and the volume of media adjusted to attain a 4.5 × 10^4^ cells/mL concentration. One milliliter of each suspension was then transferred to a 1.5-mL Eppendorf tube, centrifuged at 500 *g* for 5 minutes at 4°C, and re-suspended in 50 μL of ice-cold PBS. Following a further centrifugation under the same conditions, the pellet was re-suspended in 50 μL of a transposition mix containing TD Buffer (25 μL), TDE1 (2.5 μL), nuclease free water (22 μL), and digitonin 1% (0.5 μL) (Illumina FC-121-1030). Samples were incubated with the transposition mix for 30 minutes, undergoing a brief vortex every 10 minutes. Immediately after incubation, transposed samples were purified using the MinElute PCR Purification kit (Qiagen, 28004) as per the manufacturer’s instructions and PCR amplified with Nextera sequencing adaptors for 11–13 cycles. Right-side-size SPRI bead selection with a ratio of ×0.5 was then performed to remove fragments under 100 bp and over 1000 bp in size (Beckman, B23317). Quality control was performed using a Qubit High Sensitivity DNA assay (ThermoFisher Q32851) and Agilent Technologies Bioanalyzer, to check for nucleosome banding within the generated libraries. Libraries were sequenced using 75-bp paired-end-reads on 2 lanes of an Illumina HiSeq 4000 instrument.

### RNA-seq data analysis

Sequenced reads were assessed for quality using FastQC^42^ and trimmed of overrepresented sequences and adapter contamination from the Smart-Seq2 library preparation. Post-trimming, reads were then aligned to the human genome (hg19) using the HISAT2 aligner with default parameters.^43^ The generated Sequence Alignment Map (SAM) files were converted to Binary Alignment Maps (BAMs) using SAMtools,^44^ and matched BAM files from both lanes were merged using Picard. Merged BAMs were assessed within SeqMonk using the RNA-seq quantitation pipeline. Within the SeqMonk graphical user interface, DESeq2 was used to identify differentially expressed genes (>2-fold) using multiple correction testing (FDR ≤0.05).^45^ Heatmaps of differentially expressed genes were generated using Heatmapper.ca using an average linkage method for hierarchical clustering. Biological Process GO of upregulated genes in each cell type was performed using the “Statistical Overrepresentation Test” in Panther on default settings including an FDR test correction.^18^ RNA-seq data were compared with genes identified in a study by Hakelien et al.^22^ The gene list itself was obtained from a later publication by Tarkkonen et al.^46^

Ingenuity Pathway Analysis upstream regulator analysis using genes differentially expressed between DP OMSW and PFi OMSW was used to predict upstream regulators and help select factors from motif analysis (see ATAC-seq data analysis). Here, only transcription regulators (BH-corrected p-value <0.05) were assessed, with other IPA molecules excluded.

### ATAC-seq data analysis

Sequenced raw reads in FastQ format from lanes 1 and 2 were merged and processed using the esATAC R script, an all-in-one ATAC-seq analysis pipeline.^47^ Summary statistics about each replicate can be found in Table S1. Within the esATAC pipeline, AdapterRemoval^48^ was used for adapter trimming and alignment to the human genome (hg19) was performed using Bowtie2 with ATAC-seq bespoke parameters.^49^ Sorting of reads, duplicate removal, and read shifting due to Tn5 insertion were performed within the esATAC pipeline. The identification of open chromatin peaks was performed using F-seq,^50^^,^^51^ followed by peak annotation using ChIPseeker.^52^ Within the esATAC pipeline, comparative analysis between the cell types in each condition was then performed to identify condition-specific peaks. A condition-specific peak can be defined as a peak that has been called in 1 cell type that is not present in the exact matching genomic location in other cell types within the comparison. Motif analysis was performed on condition-specific peaks using HOMER with the given parameters: masked repeats, size given, and reference genome hg19.^53^ Known motif enrichment and de novo motif enrichment were both carried on all conditions. Known motif enrichments were compared between DP and PFi within specific conditions. Motifs were considered to be enriched if motif was present in more than 5% of target sequences, FC vs background >1.2, and p-value <1 × 10^-30^. Enriched factors were then confirmed in de novo analysis to reduce the risk of false positives due to biasing. IPA upstream regulator analysis (see above) was further used to corroborate enriched motifs identified using HOMER. The annotated lists containing all of the condition-specific peak locations were then filtered for gene promoters (esATAC default settings of ±1000 bp around the TSSs were used). Any genes that contained a condition-specific peak within its promoter range in both cell types of interest were discarded. Using the remaining genes that contained a condition-specific peak within their promoters, gene ontology was performed using IPA (Qiagen, Inc). The top 5 physiological system development and functions for each condition, generated through a core analysis, were reported as a bubble graph with significance presented on a -log scale and the size of the bubble representative of the number of genes for that category. Last, lists of RNA-seq differentially expressed genes and ATAC gene promoters were cross-compared to determine the percentage of promoters open with genes upregulated. The Integrative Genomics Viewer was used to view sequenced datasets.^54^^,^^55^

### ATAC-seq comparative analysis

The ATAC-seq promoter gene lists from each comparison, inclusive of gene promoters that contained a condition-specific peak in both cell types, were compared and visualized using UpSet^24^ to assess variation in chromatin peaks across all the conditions and cell types. Using the InteractiVenn online tool^23^ and the same datasets used in UpSet intersection visualization, gene lists containing comparison unique genes were exported and assessed in IPA using core analysis.

### mRNA validation

RT-qPCR was performed to validate RNA-seq pipeline analysis. Glyceraldehyde-3-phosphate dehydrogenase (GAPDH) was used as the housekeeping control. Shortwave-exposed cells were harvested for RNA isolation. The cells were homogenized by centrifugation through Qiashredders, and RNA was isolated using a commercially available kit as per the manufacturer’s instructions (Qiagen, RNeasy Plus Micro kit, 74034). A total of 100 ng of isolated RNA was then synthesized into complementary DNA by reverse transcription (cDNA; SuperScript III Reverse Transcriptase, ThermoFisher, 18080-093). To quantify mRNA expression, qPCR was performed using a StepOnePlus system (Applied Biosystems). cDNA was combined together with H_2_O and Syber reagents (PowerUp Sybr Green Master Mix, ThermoFisher, A25779). RT-qPCR reactions were performed in quadruplicate. The thermocyclic conditions included an initial hold stage of 50°C for 2 minutes, then 95°C for 2 minutes, followed by 40 cycles of 95°C for 15 seconds and 60°C for 1 minute. Results were normalized to GAPDH and calculated using the ΔΔCt method. Statistical analyses were performed in GraphPad Prism (version 6). Statistical differences were assessed using a 2-tailed Student’s *t* test. A p-value ≤.05 was deemed to be statistically significant. Validated RT-qPCR genes from RNA-seq pipeline analyses are shown in Supplementary Figure S7. The GAPDH primer was taken from,^56^ while other primers were designed against, sequences in the University of California Santa Cruz (UCSC) database (Supplementary Table S4).

### Ethics approval and consent to participate

The DP and PFi were isolated from human tissues, collected from patients who gave their written informed consent using Joint Research Compliance Office–approved consent forms (Imperial College Research Ethics Committee reference: 17IC3726). Tissue was held in the Imperial College Healthcare Tissue Bank (ICHTB) under the Human Tissue Authority license 12275 and used in the ICHTB-approved project R15055-1A. The study itself, and experimental protocols associated with the study, were approved by the Joint Research Compliance Office. Methods were performed in accordance with the relevant guidelines and regulations.

## Acknowledgments

The authors acknowledge support from the Centre for Blast Injury Studies (Imperial College London) for giving them access to their bespoke shock tube.

## Author contributions

Niall J Logan (Formal analysis, Investigation, Writing—original draft), Krystyna L Broda (Formal analysis, Investigation, Writing—review & editing), Nikolaos Pantelireis (Formal analysis, Investigation, Writing—review & editing), Greg Williams (Resources, Writing—review & editing) and Claire A Higgins (Conceptualization, Funding acquisition, Resources, Supervision, Writing—review & editing).

## Funding

This project was funded with a grant from the Medical Research Council (M01858X/1) to C.A.H. (https://mrc.ukri.org/). The funders had no role in study design, data collection and analysis, decision to publish, or preparation of the manuscript.

## Conflicts of interest

The authors declare they have no competing interests.

## Data availability

Both ATAC-seq and RNA-seq data are available on the NCBI Sequencing Read Archive (SRA). ATAC-seq (SRA: SRP192978, https://trace.ncbi.nlm.nih.gov/Traces/sra/?study=SRP192978). RNA-seq (SRA: SRP287348, https://trace.ncbi.nlm.nih.gov/Traces/sra/sra.cgi?study=SRP287348).

## Supplementary Material

Supplementary_files_JBMR_plus_Revisions_NoTrackChanges_ziae025

Table_S2_ziae025

Table_S3_revised_ziae025
