# Supplementary material for: Chromatin accessibility profiling reveals that human fibroblasts respond to mechanical stimulation in a cell-specific manner
Source: JBMR Plus. 2024 Feb 29;8(5):ziae025. doi: 10.1093/jbmrpl/ziae025 (PMC11055960; doi:10.1093/jbmrpl/ziae025)
Supplement: Supplementary_files_JBMR_plus_Revisions_NoTrackChanges_ziae025 [file supplementary_files_jbmr_plus_revisions_notrackchanges_ziae025.docx]

**SUPPLEMENTARY INFORMATION**

**Figures and Tables**

**
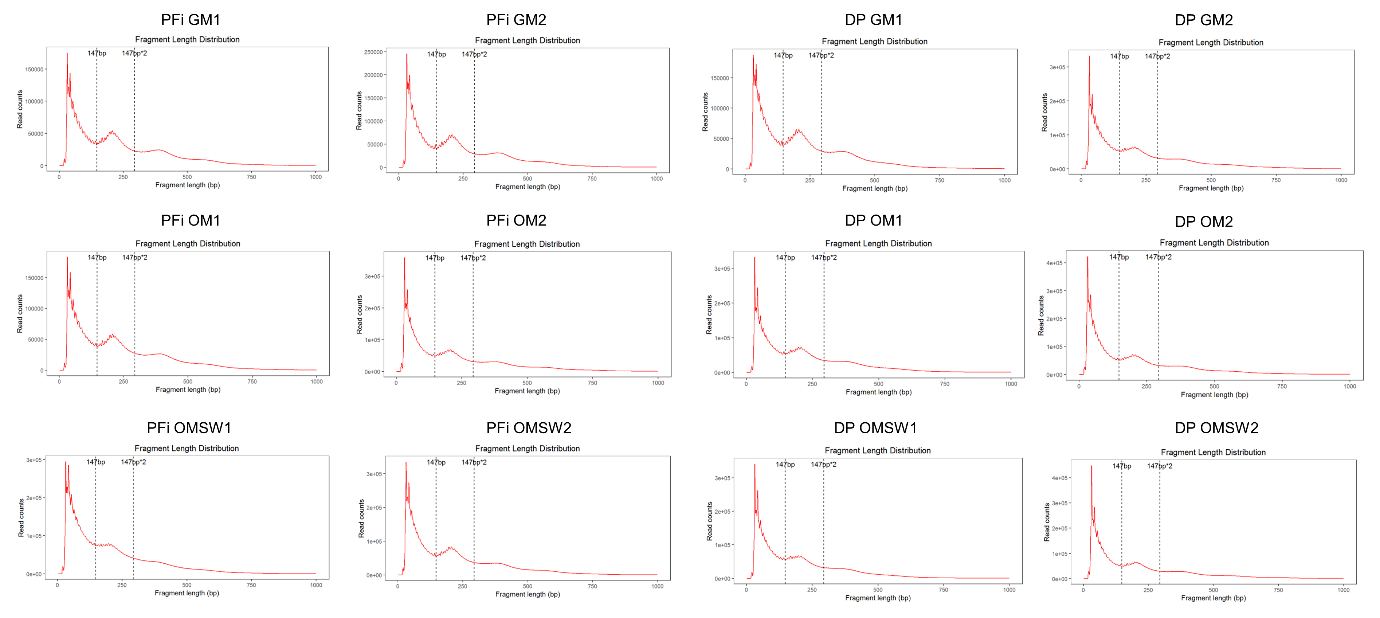
**

**Figure S1: ATAC-seq nucleosome banding**. Each prepared library is shown, with clear banding indicating good quality libraries.


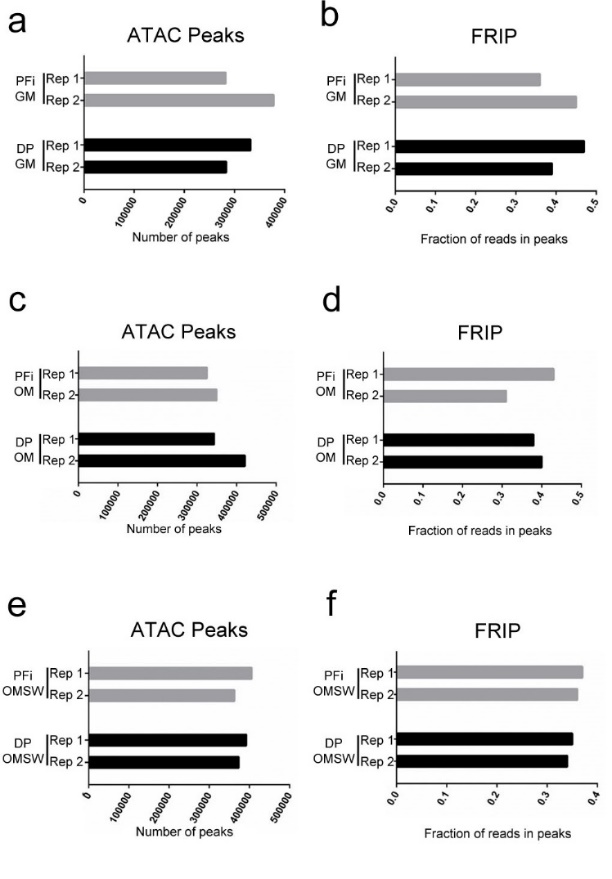


**Figure S2: ATAC peaks and FRiP scores for all samples.** a) Number of ATAC peaks in individual biological replicates in GM; b) FRiP scores from individual biological replicates in GM; c) Number of ATAC peaks in individual biological replicates in GM; d) FRiP scores from individual biological replicates in OM; e) Number of ATAC peaks in individual biological replicates in GM; f) FRiP scores from individual biological replicates in OMSW.


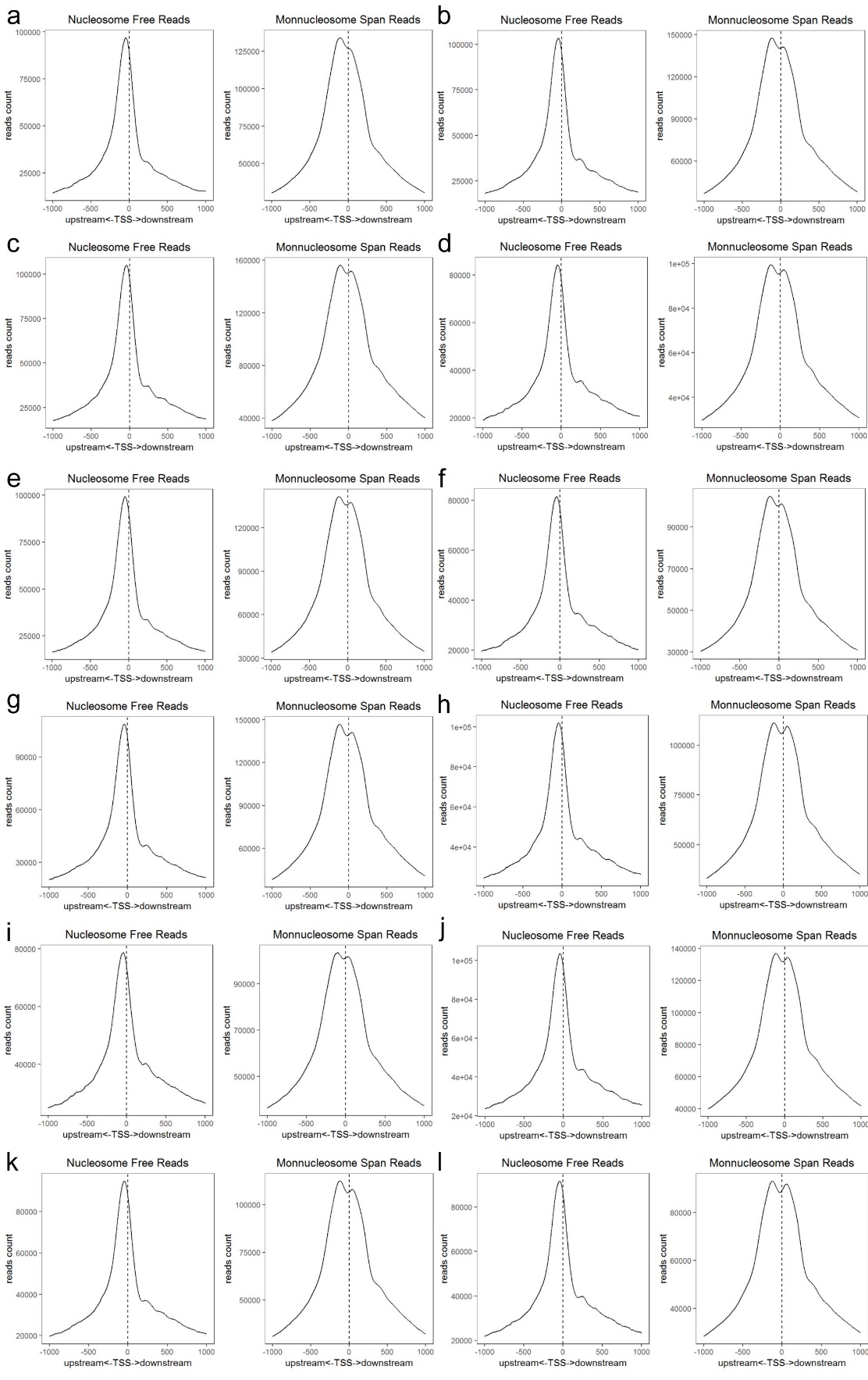


**Figure S3. TSS Enrichment peaks.** The nucleosome free reads (<100bp) and mononucleosome span reads (180-247bp) enrichment around transcription start sites (TSS) are shown for a) PFI GM1, b) PFI GM2, c) DP GM1, d) DP GM2, e) PFI OM1, f) PFI OM2, g) DP OM1, h) DP OM2, i) PFI OMSW1, j) PFI OMSW2, k) DP OMSW1, l) DP OMSW2.


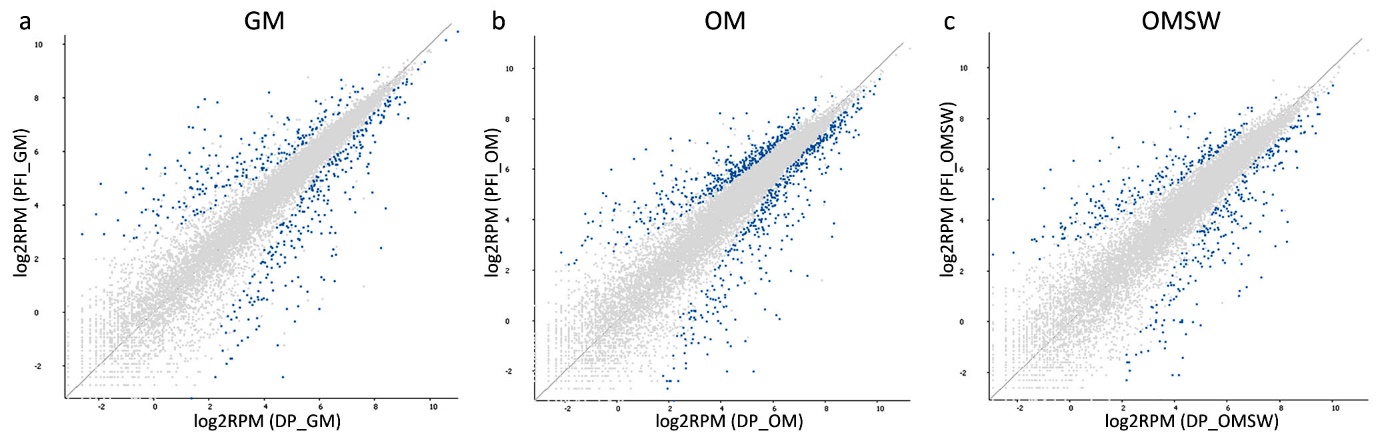


**Figure S4. RNA-seq plots.** Scatter plots showing distribution of differentially expressed genes (blue) and all genes (grey) in DP and PFI grown in a) GM, b) OM and c) OMSW.

**
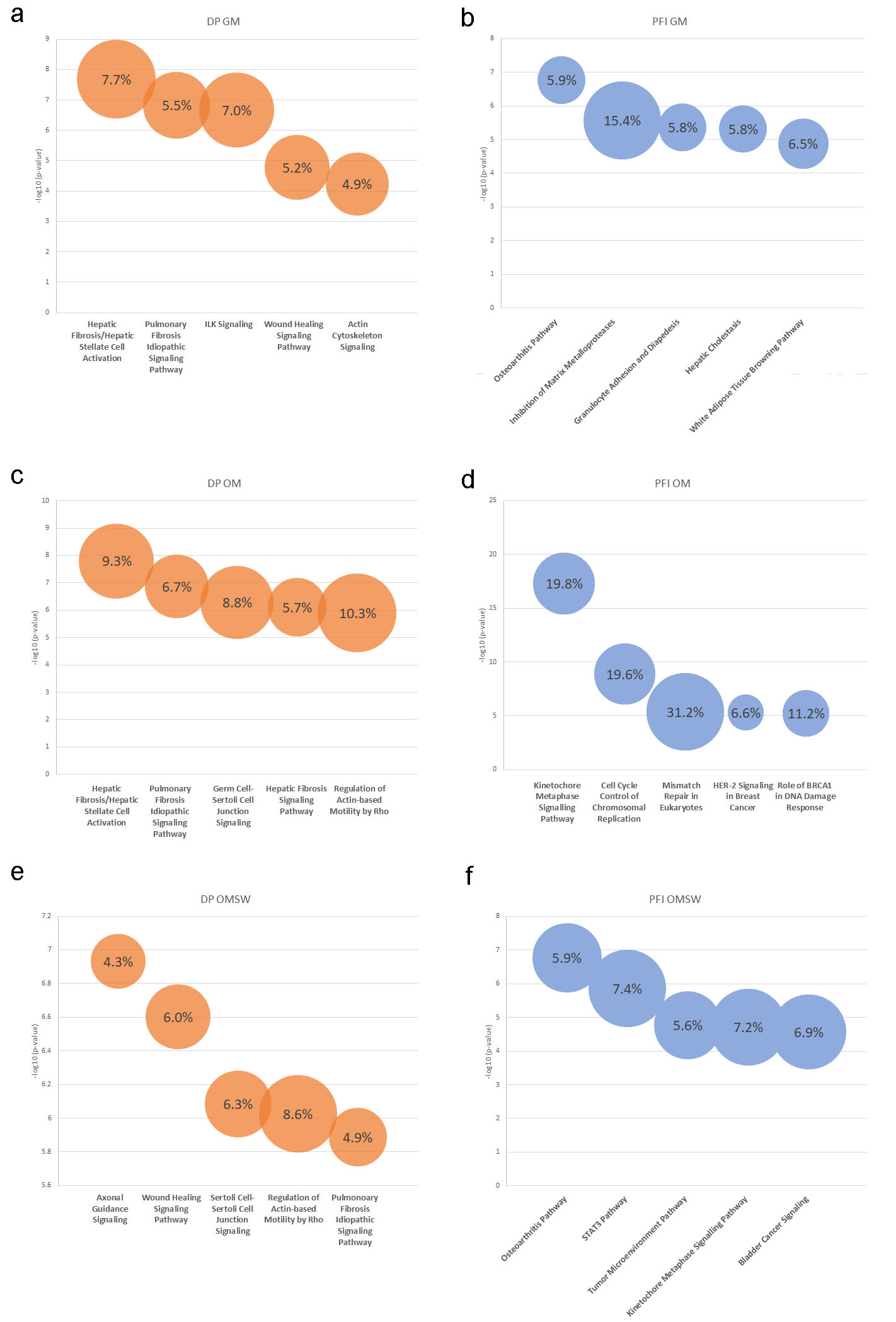
**

**Figure S5. Canonical pathway analysis from IPA.**  Top 5 canonical pathways for RNA-seq gene lists with % overlap with canonical pathway represented by the size of the bubble and -log p-value shown on the y-axis, for DP in GM (a), PFI in GM (b), DP in OM (c), PFI in OM (d), DP in OMSW (e) and PFI in OMSW (f).

**
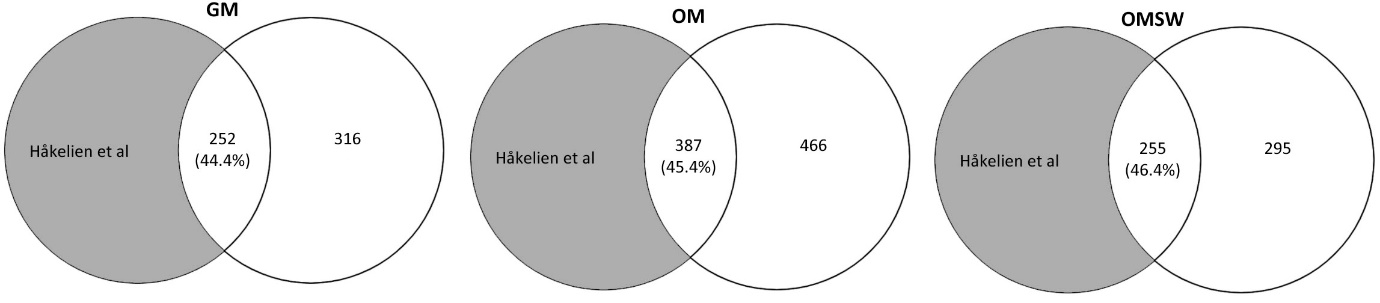
**

**Figure S6: Venn diagrams.** Venn diagrams showing the % overlap of RNA-seq gene lists in the current study (GM, OM and OMSW), with a gene list published by [Håkelien](javascript:;) et al, 2014.


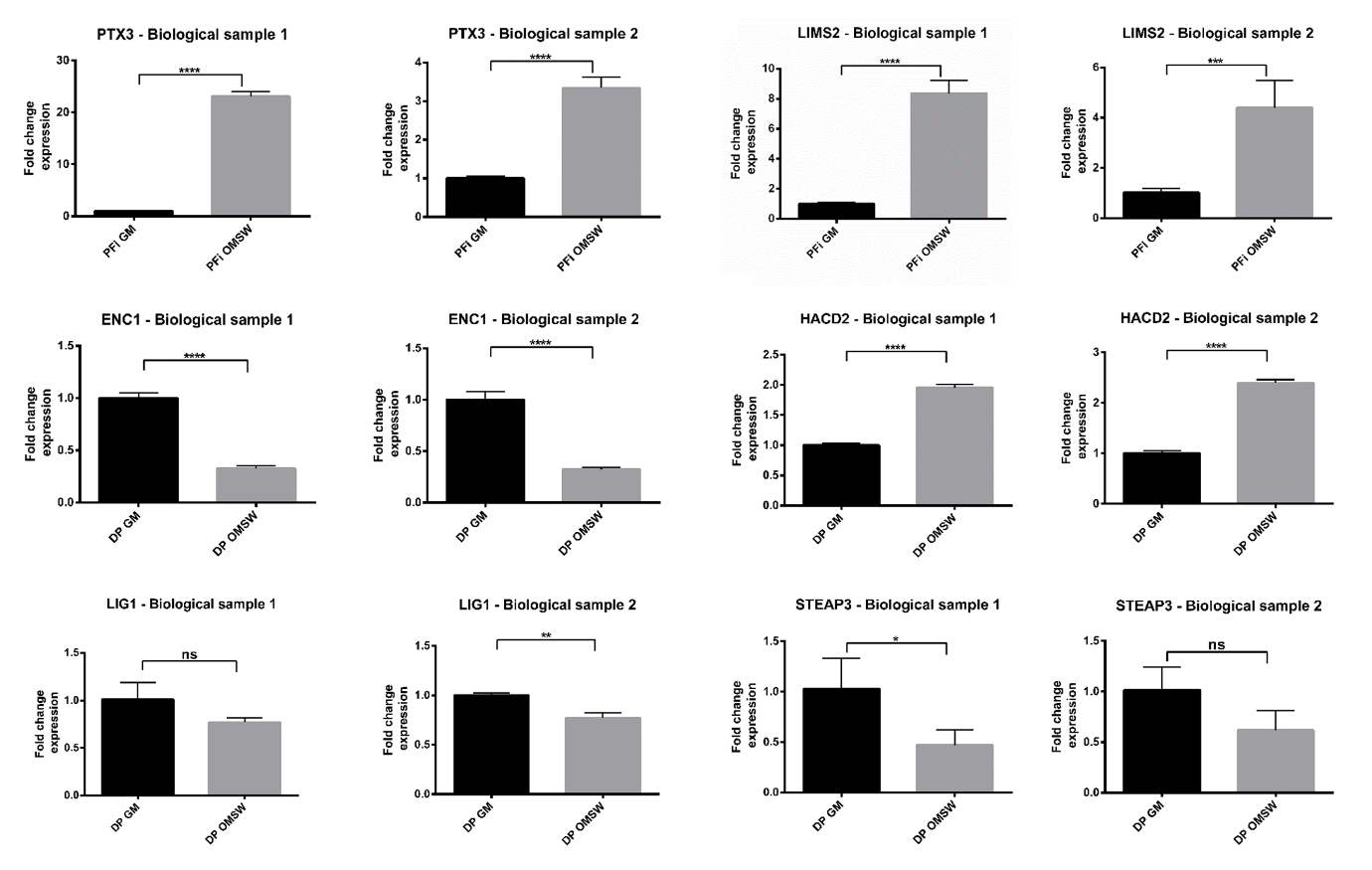
**Figure S7: RT-PCR data validating RNA-seq results.**  Gene names are shown above graphs, in addition to the biological replicate in which the validation was performed. In all cases, the trend was the same as that observed in the RNA-seq results. Significance shown on graphs calculated using technical repeats (ns=p>0.05, *=p<0.05, **=p<0.01, ***=p<0.001, ****=p<0.0001).

|  | **Total Reads** | **Survived Trimming** | **Total Mapped** | **Non-mitochondrial** | **After duplicate removal** | **Non Redundant Fraction** |
| --- | --- | --- | --- | --- | --- | --- |
| **PFI GM1** | 40243082 | 40224372 | 37295754 | 34459733 | 19271025 | 0.64 |
| **PFI GM2** | 44876270 | 44866417 | 41611047 | 39970916 | 25044847 | 0.72 |
| **DP GM1** | 35424211 | 35394800 | 33707290 | 32855555 | 23093363 | 0.81 |
| **DP GM2** | 49848939 | 49787480 | 47182570 | 44376116 | 27702733 | 0.74 |
| **PFI OM1** | 36899791 | 36853425 | 34511563 | 33411664 | 21664878 | 0.75 |
| **PFI OM2** | 49932791 | 49915178 | 46921354 | 45213561 | 28367230 | 0.75 |
| **DP OM1** | 43514853 | 43491510 | 41551282 | 40410356 | 27887282 | 0.82 |
| **DP OM2** | 44718296 | 44688475 | 42902713 | 42089528 | 27887282 | 0.87 |
| **PFI OMSW1** | 54420677 | 54396021 | 51602486 | 50456372 | 33565594 | 0.8 |
| **PFI OMSW2** | 48784813 | 48773017 | 46279484 | 45601722 | 31974191 | 0.84 |
| **DP OMSW1** | 43392671 | 43368185 | 41578060 | 40043848 | 27317727 | 0.82 |
| **DP OMSW2** | 46755837 | 46709857 | 44649814 | 43108646 | 29244793 | 0.82 |

**Table S1:** Summary and filtering statistics taken from the esATAC report for each sample

**Table S2 (excel file) – Known motif enrichment for DP and PFI in OMSW, OM, and GM.** Tables show filtered list of enriched motifs ranked on DP OMSW p-value. Motifs were filtered as follows: motifs had to be present in >5% of target sequences, show >1.2 fold enrichment in DP, and <1.2 fold enrichment in PFI. Extremely low p-values are expected in this type of analysis for significant motifs, with < 1e-50 recommended by the authors of the software.

**Table S3 (excel file) – IPA predicted upstream regulators for genes upregulated in DP and PFI OMSW.** Factors shown are termed “transcription regulators” in IPA with BH-corrected P-value <0.05. Activation scores are indicated; factors were considered to activated or inhibited if the z-score was >2 or <2 respectively. Predicted factors that appeared in PFI OMSW upregulated genes are noted.

| **Primer name** | **Sequence (5’ to 3’)** | **Annealing temperature (°C)** |
| --- | --- | --- |
| GAPDH-F | CGTCTTCACCACCATGGAGA | 60 |
| GAPDH-R | CGGCCATCACGCCACAGTTT |  |
| PTX3-F | CATCTCCTTGCGATTCTGTTTTG | 60 |
| PTX3-R | CCATTCCGAGTGCTCCTGA |  |
| LIMS2-F | GCACCGGCACTATGAGAAGAA | 60 |
| LIMS2-R | ACGGGCTTCATGTCGAACTC |  |
| ENC1-F | GCCAGCCATCTATCTCATGGA | 60 |
| ENC1-R | GGTTACCACACCGTCATTCTG |  |
| HACD2-F | GCAGTAACACATAGCGTCAAAGA | 60 |
| HACD2-R | TGATGAGGTAAGGCAGATGGTT |  |
| LIG1-F | GCCCTGCTAAAGGCCAGAAG | 60 |
| LIG1-R | CATGGGAGAGGTGTCAGAGAG |  |
| STEAP3-F | CAGCCCTATGTGCAGGAAAG | 60 |
| STEAP3-R | GCAAGTACACGAGTGACAGCA |  |

**Table. S4. Primer sequences used in validation.** GAPDH taken from Farshdousti Hagh et al [47]. Other primers designed against sequences in the UCSC database
